# Long-term disease-free survival after bilateral video-assisted thoracoscopic resection of multiple pulmonary metastases from endometrial stromal sarcoma: An 8-year follow-up case report

**DOI:** 10.1016/j.gore.2025.101954

**Published:** 2025-09-11

**Authors:** Eitetsu Koh, Yasuo Sekine, Tadao Nakazawa, Kenzo Hiroshima

**Affiliations:** a Department of Thoracic Surgery, Tokyo Women’s Medical University Yachiyo, Medical Center, Japan; b Department of Pathology, Tokyo Women’s Medical University Yachiyo, Medical Center, Japan

**Keywords:** Endometrial stromal sarcoma, Pulmonary metastases, Bilateral lung lesions, Video-assisted thoracoscopic surgery, Hormonal therapy, Long-term survival

## Abstract

**Background:**

Low-grade endometrial stromal sarcoma (LG-ESS) is a rare uterine malignancy prone to late recurrence and distant metastasis, most commonly to the lungs. Bilateral multiple pulmonary metastases are exceedingly uncommon, and optimal management remains unclear.

**Case presentation:**

A 51-year-old woman underwent total abdominal hysterectomy for LG-ESS at age 43. Eight years later, routine chest radiography revealed multiple bilateral pulmonary nodules. Computed tomography showed three well-circumscribed nodules (7–9 mm) in the left S4, left S8, and right S8 segments. Positron emission tomography showed minimal fluorodeoxyglucose uptake. Bilateral video-assisted thoracoscopic wedge resections were performed, achieving complete removal of all lesions. Histopathology confirmed metastatic LG-ESS (CD10/ER/PR positive). The patient received oral medroxyprogesterone acetate for five years. The therapy was discontinued following subarachnoid hemorrhage due to a ruptured posterior-circulation intracranial aneurysm and treated by surgical clipping; she has no residual neurological deficits. She remains disease-free a total of eight years after metastasectomy.

**Conclusion:**

Complete resection of bilateral multiple pulmonary metastases from LG-ESS, combined with hormonal therapy, can achieve durable long-term disease control in carefully selected patients.

## Highlights

• Endometrial stromal sarcoma can metastasize to the lungs years after hysterectomy.
• Bilateral multiple pulmonary metastases from ESS are extremely rare.
• Complete resection via VATS achieved in a patient with bilateral lung lesions.
• Five years of adjuvant progestin therapy followed by cessation due to subarachnoid.
• hemorrhage. Eight-year disease-free survival after metastasectomy without recurrence.

## Graphical abstract

**Figure:**
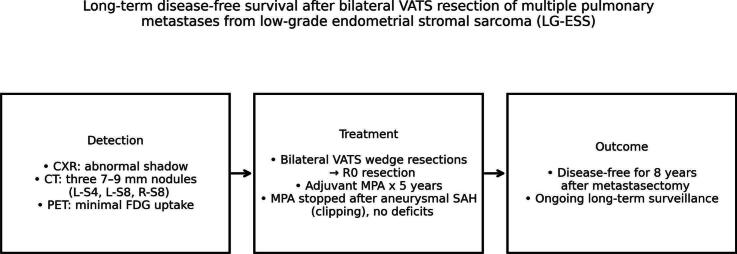

## 1 Introduction

Low-grade endometrial stromal sarcoma (LG-ESS) is a rare uterine malignancy, accounting for less than 1 % of all uterine cancers and approximately 10–15 % of uterine sarcomas. It is characterized by indolent growth but a propensity for late recurrence and distant metastasis, most commonly involving the lungs. Pulmonary metastases may occur several years after the initial diagnosis, even in patients who underwent complete hysterectomy, reflecting the tumor’s unique biological behavior. Surgical resection of metastatic lesions, when feasible, has been reported to offer both diagnostic confirmation and potential survival benefit in selected patients.

Bilateral multiple pulmonary metastases from LG-ESS are exceedingly uncommon, and only a limited number of cases have been documented in the literature. Most reported cases involve solitary or unilateral lung nodules, while bilateral lesions present unique diagnostic and therapeutic challenges. Optimal management strategies for such cases remain unclear, particularly regarding the role and timing of surgical intervention. Furthermore, there is a paucity of data describing minimally invasive approaches, such as video-assisted thoracoscopic surgery (VATS), in this setting.

The purpose of this report is to describe the clinical course, surgical management, and pathological findings of this rare presentation, and to review the relevant literature regarding pulmonary metastases from LG-ESS. By detailing the diagnostic workup, surgical approach, and postoperative outcomes, we aim to contribute to the growing body of evidence supporting the role of surgical resection, even in cases with bilateral multiple lesions. This case also highlights the potential utility of adjuvant hormonal therapy in the postoperative setting for LG-ESS with pulmonary metastases.

## 2 Case presentation

**Fig. 1:**
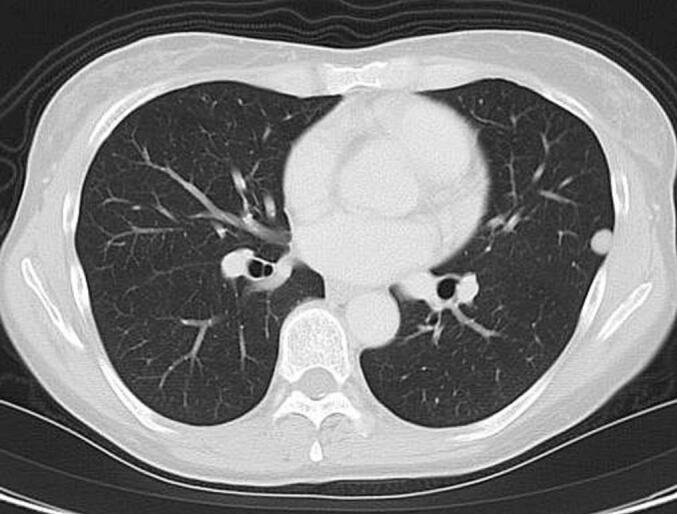

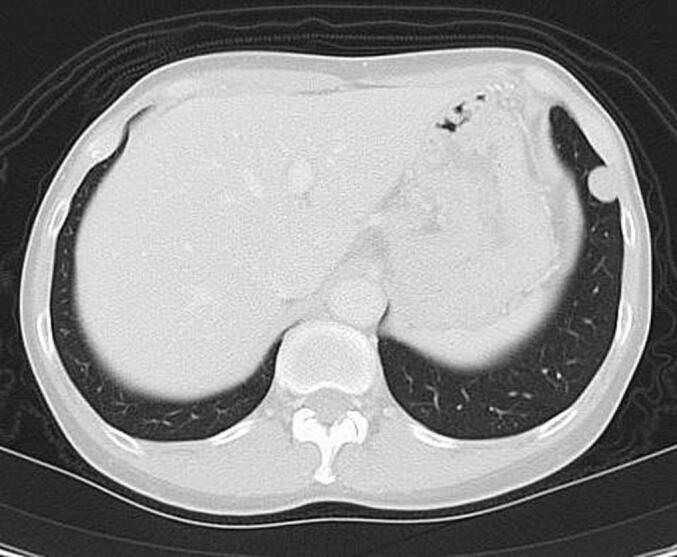

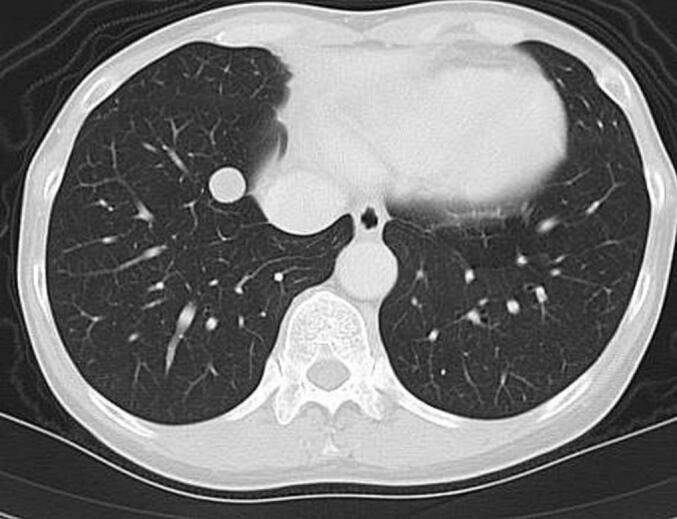
.

A 51-year-old woman had undergone total abdominal hysterectomy at the age of 43 for low-grade endometrial stromal sarcoma. She had been regularly followed without evidence of recurrence. Eight years after the initial surgery, a routine workplace health examination revealed an abnormal shadow on chest radiography. She was referred to our respiratory medicine department for further evaluation. Chest computed tomography (CT) demonstrated a 7-mm well-circumscribed, homogeneous nodule in the left S4 segment, a 9-mm nodule in the left S8 segment, and a 9-mm nodule in the right S8 segment. The nodules exhibited smooth margins and homogeneous density (Fig. 1). Laboratory data, including complete blood count and serum biochemistry, were within normal limits. Positron emission tomography (PET) revealed no significant fluorodeoxyglucose uptake, with an early-phase SUVmax of 0.9 and delayed-phase SUVmax of 1.4, findings suggestive of metastatic pulmonary tumors.

**Fig. 2:**
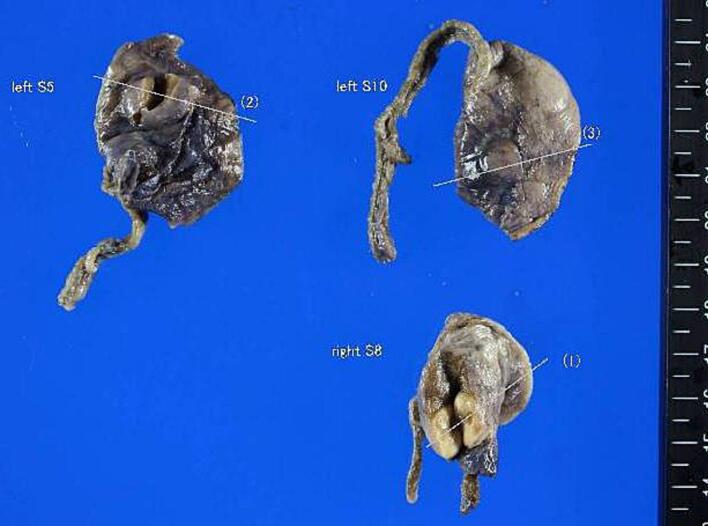
.

The patient subsequently underwent bilateral video-assisted thoracoscopic surgery (VATS) for diagnostic and therapeutic purposes. Three wedge resections were performed: one each from the right S8 and left S4 segments, and one from the left S8 segment. Intraoperatively, all nodules were well-demarcated, firm, and located within the peripheral lung parenchyma without pleural invasion. No other intrathoracic abnormalities were identified. The resected specimens measured 7–9 mm in greatest diameter, with a tan-white, homogeneous cut surface on gross examination (Fig. 2). The patient tolerated the procedure well, with no intraoperative complications.

**Fig. 3:**
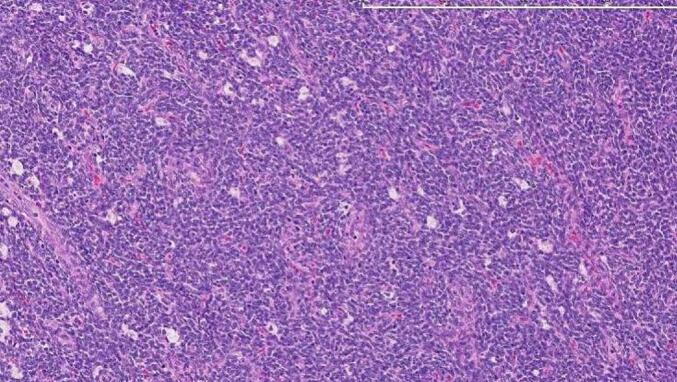
.

Histopathological examination revealed a proliferation of small, uniform spindle-shaped cells with scant cytoplasm, arranged in a storiform and whorled pattern, resembling proliferative-phase endometrial stroma. Mitotic figures were infrequent, and there was no evidence of significant nuclear atypia or necrosis. Immunohistochemical analysis showed strong positivity for CD10, estrogen receptor (ER), and progesterone receptor (PR), supporting the diagnosis of metastatic low-grade endometrial stromal sarcoma. The surgical margins were free of tumor in all resected specimens (Fig. 3).

She subsequently commenced adjuvant hormonal therapy with oral medroxyprogesterone acetate at a daily dose of 200 mg. The patient continued this regimen for five years without evidence of recurrence. The therapy was discontinued after she developed a subarachnoid hemorrhage that occurred in the setting of long-standing hypertension with hyperglycemia. The hemorrhage was due to a ruptured posterior-circulation intracranial aneurysm and was treated by surgical clipping. The event was not considered related to medroxyprogesterone acetate by the treating neurosurgical team, and the patient has no residual neurological deficits. She has since remained disease-free for an additional three years without hormonal therapy, for a total of eight years of recurrence-free survival after metastasectomy.

Follow-up and surveillance. After metastasectomy, surveillance consisted of clinical examination every 3–6 months for 3 years, every 6–12 months for years 4–5, and annually thereafter, with CT of the chest/abdomen/pelvis every 6 months for 3 years and annually thereafter (transitioning to chest X-ray after year 5 to limit radiation), and symptom-triggered imaging as needed.

## 3 Discussion

This case illustrates a rare presentation of bilateral multiple pulmonary metastases from LG-ESS occurring eight years after primary hysterectomy. LG-ESS is known for its indolent clinical course but high propensity for late recurrence and distant metastasis, most commonly to the lungs. While solitary or unilateral pulmonary lesions have been more frequently described, bilateral multiple metastases are exceptionally rare. In our patient, complete surgical resection of all detectable pulmonary lesions was achieved via a minimally invasive video-assisted thoracoscopic approach, followed by adjuvant hormonal therapy. The favorable long-term outcome in this case supports the consideration of surgical intervention as part of a multidisciplinary strategy for selected patients with resectable pulmonary metastases from LG-ESS.

Surgical resection has been reported as a potentially beneficial treatment for selected patients with pulmonary metastases from LG-ESS, particularly when the disease is limited and complete removal is feasible. Previous reports suggest that metastasectomy may prolong survival and provide long-term disease control in indolent tumors such as LG-ESS, especially in cases with hormone receptor–positive disease. However, most published cases have described solitary or unilateral lesions, and evidence supporting surgical management of bilateral multiple metastases remains sparse. Our case demonstrates that even with bilateral involvement, minimally invasive surgery such as VATS can achieve complete resection with low morbidity, thereby facilitating early recovery and timely initiation of adjuvant therapy.

Activity of endocrine therapy and rationale for surgery-first. LG-ESS is typically hormone-responsive. Retrospective series report high activity for aromatase inhibitors in recurrent or residual LG-ESS, with robust disease control across small studies. When complete resection of recurrent or metastatic disease is feasible, metastasectomy is recommended/considered to provide diagnostic confirmation and durable control with low morbidity, with endocrine therapy instituted post-operatively. In this case, three small peripheral nodules were bilaterally accessible by VATS, allowing R0 resection and timely adjuvant therapy.

Endocrine regimens and dosing (typical). Progestins such as medroxyprogesterone acetate (approximately 250–500 mg/day) or megestrol acetate (approximately 160–320 mg/day), aromatase inhibitors such as letrozole (2.5 mg/day), anastrozole (1 mg/day), or exemestane (25 mg/day), and GnRH agonists such as leuprolide (3.75 mg intramuscularly every 28 days) are commonly used; selection is individualized. Tamoxifen and exogenous estrogen are generally avoided in LG-ESS.

The present case underscores the importance of a multidisciplinary approach in the management of metastatic LG-ESS, integrating surgical resection and systemic therapy. Although LG-ESS is rare, its tendency for late recurrence and distant spread necessitates long-term surveillance. Our experience suggests that even in the setting of bilateral multiple pulmonary metastases, aggressive local control with minimally invasive surgery can be feasible and may offer a survival advantage when combined with appropriate adjuvant hormonal therapy. Further accumulation of similar cases and prospective studies are needed to better define patient selection criteria, optimal timing of surgery, and the role of adjuvant systemic therapy.

In summary, this case highlights the successful management of bilateral multiple pulmonary metastases from LG-ESS through complete resection using a minimally invasive surgical approach combined with adjuvant hormonal therapy. The rarity of such presentations and the paucity of robust data make individualized treatment planning essential. Our findings support the potential benefit of aggressive local therapy in selected patients with resectable disease. Continued reporting of similar cases will be critical to refining treatment strategies and improving outcomes for patients with metastatic LG-ESS.

## 4 Conclusion

This case demonstrates that complete surgical resection of bilateral multiple pulmonary metastases from LG-ESS, combined with adjuvant hormonal therapy, can achieve long-term disease control. Our patient has remained disease-free for eight years following metastasectomy, underscoring the potential durability of this treatment approach in carefully selected patients.

## Ethical approval

Written informed consent was obtained from the patient for publication of this case report and accompanying images.

## CRediT authorship contribution statement

**Eitetsu Koh:** Writing – review & editing, Writing – original draft, Visualization, Validation, Software, Resources, Project administration, Methodology, Investigation, Funding acquisition, Formal analysis, Data curation. **Yasuo Sekine:** Supervision. **Tadao Nakazawa:** Supervision. **Kenzo Hiroshima:** Supervision.

## Declaration of competing interest

The authors declare that they have no known competing financial interests or personal relationships that could have appeared to influence the work reported in this paper.

